# Genome Wide Association Mapping for the Tolerance to the Polyamine Oxidase Inhibitor Guazatine in *Arabidopsis thaliana*

**DOI:** 10.3389/fpls.2016.00401

**Published:** 2016-04-05

**Authors:** Kostadin E. Atanasov, Luis Barboza-Barquero, Antonio F. Tiburcio, Rubén Alcázar

**Affiliations:** ^1^Laboratory of Plant Physiology, Department of Natural Products, Plant Biology and Soil Science, Faculty of Pharmacy, University of BarcelonaBarcelona, Spain; ^2^Centro para Investigaciones en Granos y Semillas, Universidad de Costa RicaSan José, Costa Rica

**Keywords:** guazatine, polyaminoguanidines, GWAS, natural variation, population genetics

## Abstract

Guazatine is a potent inhibitor of polyamine oxidase (PAO) activity. In agriculture, guazatine is used as non-systemic contact fungicide efficient in the protection of cereals and citrus fruits against disease. The composition of guazatine is complex, mainly constituted by a mixture of synthetic guanidated polyamines (polyaminoguanidines). Here, we have studied the effects from exposure to guazatine in the weed *Arabidopsis thaliana*. We report that micromolar concentrations of guazatine are sufficient to inhibit growth of *Arabidopsis* seedlings and induce chlorosis, whereas germination is barely affected. We observed the occurrence of quantitative variation in the response to guazatine between 107 randomly chosen *Arabidopsis* accessions. This enabled us to undertake genome-wide association (GWA) mapping that identified a locus on chromosome one associated with guazatine tolerance. *CHLOROPHYLLASE 1* (*CLH1*) within this locus was studied as candidate gene, together with its paralog *(CLH2)*. The analysis of independent *clh1-2, clh1-3, clh2-3, clh2-2*, and double *clh1-2 clh2-3* mutant alleles indicated that *CLH1* and/or *CLH2* loss-of-function or expression down-regulation promote guazatine tolerance in *Arabidopsis*. We report a natural mechanism by which *Arabidopsis* populations can overcome toxicity by the fungicide guazatine.

## Introduction

*Arabidopsis thaliana* (thereafter referred to as *Arabidopsis*) is a small weed mainly distributed in the northern hemisphere. It grows in open or recently disturbed habitats and its spread was facilitated by the expansion of agriculture (François et al., 2008). *Arabidopsis* exhibits extensive natural variation for different developmental, abiotic and biotic stress resistance traits (Koornneef et al., 2004; Alonso-Blanco et al., 2009; Atwell et al., 2010). Understanding the genetic bases for such variation enables the identification of potential mechanisms underlying local adaptation. Here, we have used genome-wide association studies (GWAS) to identify genes contributing to the natural variation in guazatine tolerance observed in this species. Multiple recombination events in the genetic history of populations produce close linkage disequilibrium (LD) of markers with causal loci for certain phenotypes. Such associations can be detected through GWAS. These type of approaches require the genetic validation of associations and have some limitations compared to, for example, QTL mapping (Korte and Farlow, 2013). In *Arabidopsis*, GWAS has been successfully applied to uncover the genetics of multiple traits (Atwell et al., 2010; Baxter et al., 2010; Li et al., 2010; Chan et al., 2011; Chao et al., 2012; Filiault and Maloof, 2012). Furthermore, the use of natural variation as source of genetic variability enables the analysis of how naturally occurring alleles evolve and may be selected (Alonso-Blanco et al., 2009).

Guazatine is a non-systemic, contact-based, aliphatic nitrogen fungicide used in agriculture that protects cereals against different diseases such as common bunt (*Tilletia* ssp.), common root rot (*Helminthosporium*), seedling blight (*Fusarium* ssp.), glume blotch (*Septoria*), and smut (*Ustilago;* Dreassi et al., 2007). In citrus fruits, guazatine also protects from infection by sour rot (*Geotrichum candidum*), green mold (*Penicillium digitatum*), and blue mold (*Penicillium italicum;* Wild, 1983). The mode of action of guazatine, at least in the ascomycete *Alternaria*, is inhibition of lipid biosynthesis and membrane destabilization (Yagura et al., 1984). The composition of guazatine is complex and constituted by a mixture of guanidated polyamines (PAs) referred to as polyaminoguanidines (PAGs). Most abundant PAGs in guazatine are diamines [octamethylenediamine H_2_N-(CH_2_)_8_-NH_2_] and triamines [iminodi(octamethylene)diamine H_2_N-(CH_2_)_8_-NH-(CH_2_)_8_-NH_2_], but also guanidated tetramines and carbamonitrile. In plants, guazatine is a potent inhibitor of PA oxidase activity (PAO) that has been extensively used to block PA oxidation or PA back-conversion in different species, thus contributing to decipher the biological functions of PAO in plants in relation to H_2_O_2_ production and ROS signaling (Federico et al., 2001; Yoda et al., 2006; Marina et al., 2008; Moschou et al., 2008; Fincato et al., 2011; Agudelo-Romero et al., 2014). The long alkyl chains, secondary amino groups and guanidine groups of PAGs constitute the structural requirements for the inhibition of PAO activity by guazatine (Cona et al., 2004). Despite its use in agriculture as fungicide, little is known about the physiological effects from long-term exposure to guazatine in weeds, such as *Arabidopsis*. We find that guazatine concentrations as low as 2.5 μM inhibit *Arabidopsis* shoot and root growth, and reduce total chlorophyll levels. We identified the occurrence of quantitative variation in response to guazatine in 107 natural *Arabidopsis* accessions from Europe and America. We performed genome-wide association mapping to determine the genetic bases for the variation observed. GWAS identified associations between guazatine tolerance and allelic variation at *CHLOROPHYLLASE 1* (*CLH1*), encoding an enzyme that catalyzes the hydrolysis of the ester bond of chlorophyll producing chlorophyllide and phytol (Hörtensteiner, 2006). *CLH1* and its paralog *CLH2*, were further validated for this association. The isolation and analysis of *chl1-2, clh1-3, chl2-2, clh2-3*, and double *clh1-2 clh2-3* mutant alleles confirmed that *CLH1* or *CLH2* loss-of-function promote guazatine-tolerance in *Arabidopsis*. We conclude that a natural mechanism occurs which provides tolerance to guazatine in natural populations, involving enzymes in the chlorophyll degradation pathway.

## Materials and methods

### Plant materials and growth conditions

Accessions used in this work were obtained from the Nottingham *Arabidopsis* Stock Centre (NASC, www.arabidopsis.info) or kindly provided by Prof. Maarten Koornneef (Max Planck Institute for Plant Breeding Research, Cologne, Germany). A complete list of *Arabidopsis* accessions, origins and accession numbers is detailed in Table S1. Seed sterilization was performed by vigorous shaking of seeds in an aqueous solution containing 30% sodium hypochlorite supplemented with 0.5% TritonX-100 for 10 min, followed by three washes with sterile deionized H_2_O. For *in vitro* culture, sterilized seeds were sown on Growth Media (GM: 0.5 x Murashige & Skoog supplemented with vitamins, 1% sucrose, 0.8% Plant Agar (Duchefa Biochemie), pH 5.7 adjusted with KOH). Seeds were stratified in the dark at 4°C during 2–4 days. Seedlings were grown under 12 h dark/12 h light cycles at 20/22°C, 100–125 μmol photons m^−2^ s^−1^ of light intensity.

### Isolation and characterization of *clh1-2, clh1-3, clh2-2, clh2-3* double *clh1-2 clh2-3* mutants

*clh1* and *clh2* T-DNA insertion mutants were obtained from NASC (*clh1-2*, N653869; *clh1-3*, N871333; *clh2-2*, N827897; and *clh2-3*, N668619). Confirmation of the T-DNA insertion position and isolation of homozygous lines was performed by PCR-based genotyping and sequencing from genomic DNA, using T-DNA (LB) primer (5′-GCG TGGACCGCTTGCTGCAACT) and gene specific primers: *clh1-2* (Fwd: 5′-TTTGTTAGTTCCTGCGACTGG and Rev: 5′-AGA GAGAGAGACGGAGGTTGG), *clh1-3* (Fwd: 5′-CACATACAACCGGCC ATAAAC and Rev: 5′-GAA AAATCAACATTCTCCCCC), *clh2-3* (Fwd: 5′-CGGATAATCTCCTTC CTCCAC and Rev: 5′-ACA AAGCCCATTCCTTGTACC), *clh2-2* (Fwd: 5′-GAGGGTGGAGAG AATTTGAGG and Rev: 5′ GTCGCCTTAAAGAAATTTGGG). Genomic DNA was extracted using DNeasy plant mini Kit (Qiagen) according to manufacturer's instructions. PCR conditions were as follows: 95°C 5 min, 30 cycles (95°C 15 s, 55°C 45 s, 72°C 2 min), 72°C 10 min.

The double homozygous *clh1-2 clh2-3* mutant was isolated by genotyping 48 F_2_ plants derived from the cross of the respective parental lines with primers described above. Expression of *CLH1* and *CLH2* was determined by RT-PCR. Briefly, total RNA isolated from 7-days old seedlings was extracted using TRIzol reagent (Invitrogen). Two micrograms of RNA was treated with DNAse I (Invitrogen) and first strand cDNA synthesized using Superscript II (Invitrogen) and oligo dT. One microliter of cDNA was used for PCR amplification of *CLH1* (Fwd: 5′-TTACATTCTTGTAGC CCCAC, Rev: 5′-GCG ACTGGATCAATTCCTAT) or *CLH2* (Fwd: GCTTATGTTGCATGTCTCT, Rev: CGAGGAGTA CCCAAATTTCT) with LA Taq DNA polymerase (Takara) using the following PCR conditions: 95°C 5 min, 30 cycles (95°C 15 s, 55°C 45 s, 68°C 1 min), 68°C 10 min.

### Guazatine treatments

Guazatine acetate was obtained from KenoGard (Stockholm). Sterilized seeds of *Arabidopsis* accessions were sown directly on GM supplemented with or without 2.5 μM guazatine. Chlorophyll levels were determined 16 days after germination.

*clh* mutants were germinated and grown on a nylon mesh (43 μm) placed on top of the GM media. Four days after germination, the nylon mesh was transferred to GM supplemented with 5 μM guazatine. Samples for chlorophyll extraction were harvested 12 days after guazatine treatment.

### Quantification of chlorophyll levels

Seedlings were harvested individually, weighted and placed in 2 ml tubes (Eppendorf safe-lock) in the presence of 100 μl of borosilicate beads (Ø 4 mm), submerged in liquid nitrogen and homogenized with Star-Beater device (VWR International). Buffered acetone (acetone/Tris-HCl 80:20 vol, pH 7.8) was added in a ratio of 1 ml per 20 mg fresh weight (FW). Samples were incubated in the dark at 70°C during 10 min and centrifuged at 12,000 rpm for 1 min. Chlorophylls were determined using UV2310 Spectrophotometer (DINKO Industries) at 663 nm for chlorophyll A and 645 nm for chlorophyll B. Chlorophyll levels were calculated according to Porra (2002).

### Transmission electron microscopy

Fifteen randomly chosen leaf segments from guazatine-treated and untreated leaves, were cut into pieces of 5 mm length and fixed in a solution of 2% glutaraldehyde in 2.5% cacodylate buffer pH 7.4 (CB) at 4°C. The segments were washed five times for 10 min in CB and post-fixed for 2 h 15 min in a solution of 1% OsO_4_ and 0.8% FeCNK (w/v). After five additional washes with distilled H_2_O at 4°C, samples were dehydrated in acetone and embedded in Spurr Low-Viscosity Embedding kit (Sigma-Aldrich). Serial ultrathin sections (60–70 nm) were obtained using an ultramicrotome (Reichert-Jung, Wien, Austria), collected on 200 mesh uncoated copper grids and stained with 2% uranyl acetate and Reynolds lead citrate. Samples were observed under a TEM Bioscan Gatan, JEOL 1010 at the Scientific and Technological Centers (CCiT) of the University of Barcelona.

### GWA mapping

GWAS was performed using the GWAPP web interface (Seren et al., 2012). Mean chlorophyll values of 107 *Arabidopsis* accessions grown under control and guazatine conditions (as indicated above), were transformed using the square root. GWAS was conducted using the accelerated mixed model (AMM), and linear regression (LM; Seren et al., 2012). To correct for multiple testing, a Bonferroni correction with a threshold of 0.5 was performed. *P*-value bias due to population stratification was evaluated with Q–Q plots. The LD was visualized in the flanking region of the *CLH1* gene (between 6.74 and 6.87 Mb on chromosome 1).

### Root and biomass measurements

For root measurements, 4-days old seedlings germinated on GM were transferred into GM plates containing 1.5 μM guazatine. Plates were placed vertically and root measurements determined after 12 days of guazatine treatment using the SmartRoot software (Lobet et al., 2011).

### Quantification of free PAs

PAs from plant material were extracted using 5% (v/v) perchloric acid (PCA, 1 ml per 200 mg of fresh weight). Samples were vortexed vigorously, incubated on ice during 5 min and centrifuged at 16,000 g 10 min at 4°C. 200 μl of the PCA supernatant were taken for dansyl derivatization and detection according to Marcé et al. (1995).

### Expression analyses

The expression of *CLH1* (*At1g19670*) and *CLH2* (*At5g43860*) in *Arabidopsis* accessions was obtained from microarray data deposited in Genevestigator under experiment IDs AT-00283 and AT-00407 (Hruz et al., 2008). Expression values from three independent biological replicates were normalized to *UBIQUITIN 10* (*AT4G05320*) and expressed relative to Col-0 accession.

### Phylogenetic and statistical analyses

DNA sequences were obtained from the 1001 Genomes project (www.1001genomes.org). NJ tree was computed using MEGA6.06. Statistical analyses were performed using *SPSS* software v.22 (IBM *SPSS Statistics*, IBM, Chicago, IL).

## Results

### Effects of long-term exposure to guazatine in *Arabidopsis*

**Figure 1:**
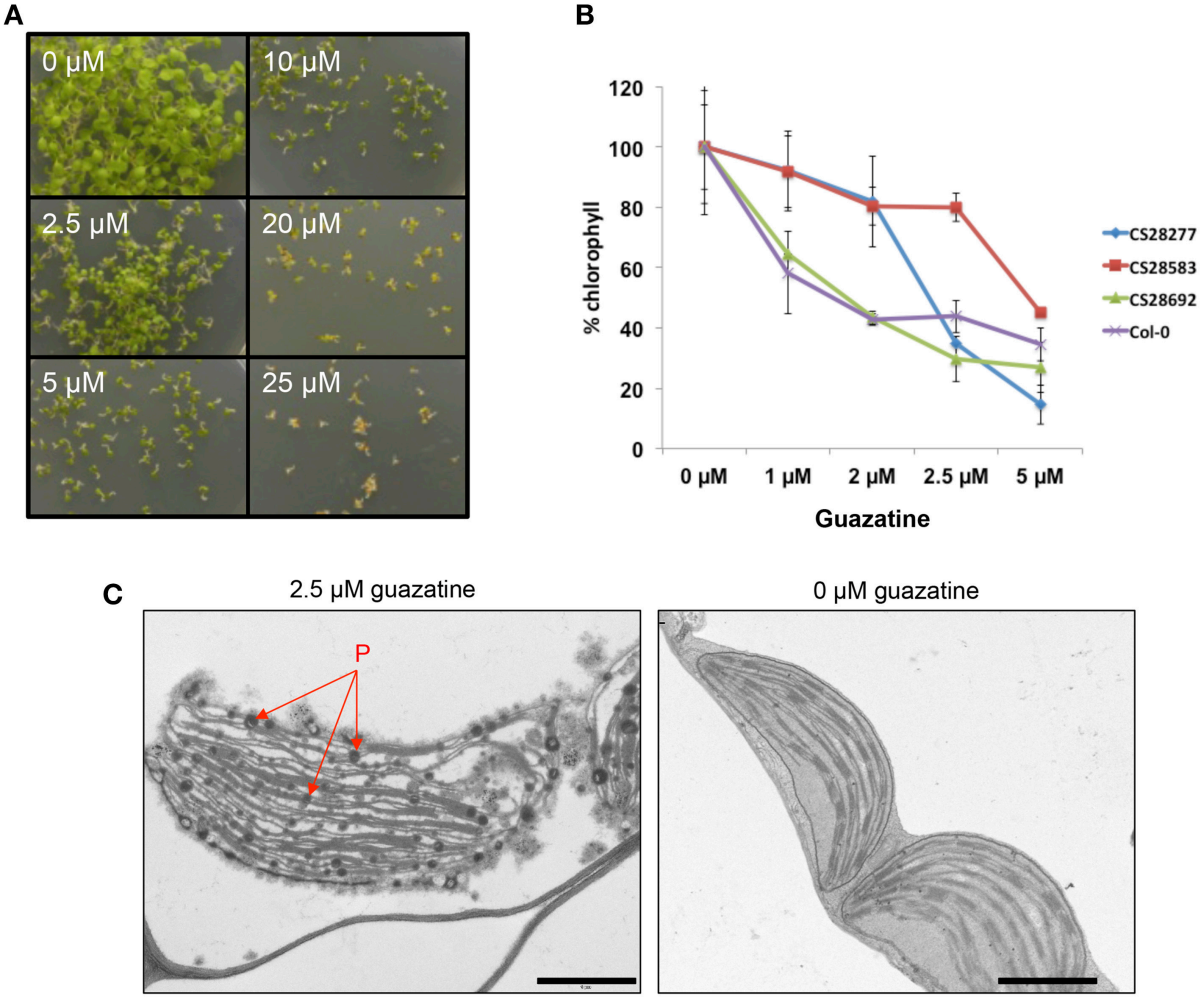
**Effects of guazatine treatment in *Arabidopsis* germination, growth and chlorophyll content. (A)** Phenotype of 12-days-old *Arabidopsis* seedlings germinated and grown under different concentrations of guazatine from 0 to 25 μM. **(B)** Quantitation of chlorophyll loss by guazatine treatment in four genetically different accessions (CS28277, Ge-1; CS28583, Old-1; CS28692, Rou-0; Col-0). Values are normalized to chlorophyll levels at 0 μM guazatine for every genotype and expressed as %. Values are the mean from at least five independent biological replicates ±SD. **(C)** TEM images of 12-days-old wild-type Col-0 seedlings treated with 2.5 μM guazatine (+) or 0 μM guazatine (−). P, plastoglobules. Scale bar: 1 μM.

Due to the use of polyaminoguanidines (guazatine) as fungicide in agriculture, we studied the effects of guazatine treatment in germination and growth of the weed *Arabidopsis*. Exposure of *Arabidopsis* (Col-0) to increasing concentrations of guazatine from 0 to 25 μM did not affect germination (Figure 1A). Conversely, treatment with guazatine inhibited growth of *Arabidopsis* seedlings, produced chlorosis and affected chloroplast integrity (Figures 1A–C). Accumulation of osmophilic bodies that resembled plastoglobules was observed in chloroplasts of guazatine-treated leaves (Figure 1C). Guazatine concentrations as low as 2.5 μM were sufficient to inhibit growth in different *Arabidopsis* accessions, whereas chlorosis exhibited a dose-dependent response depending on the accession (Figures 1A,B). We concluded that long-term exposure to μM concentrations of guazatine is detrimental for *Arabidopsis* growth and reduces chlorophyll levels, for which quantitative variation between accessions was observed.

### Quantitative variation of chlorophyll levels in response to guazatine in 107 *Arabidopsis* accessions

**Figure 2:**
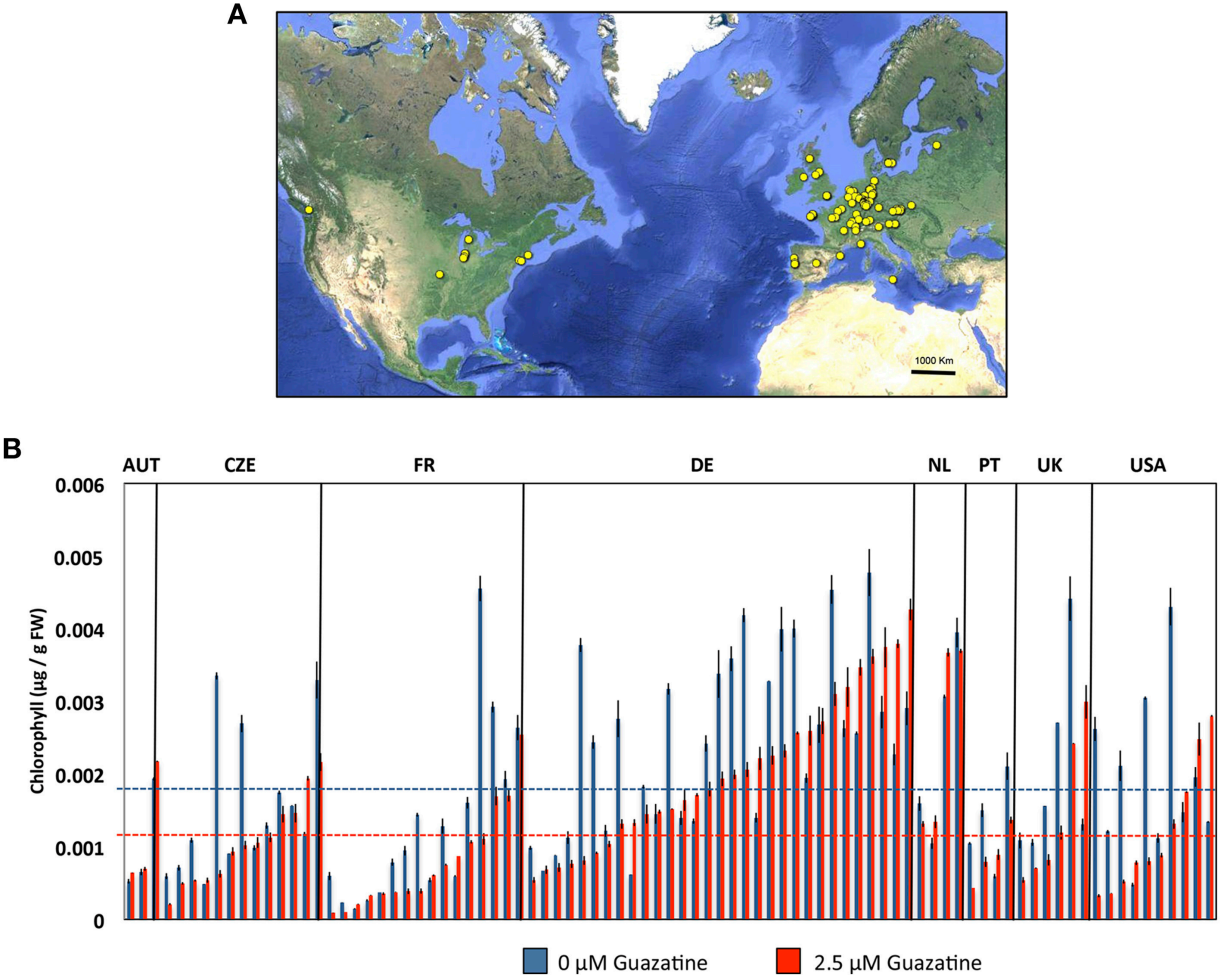
**(A)** Geographical distribution of *Arabidopsis* accessions used in this work. The origins of accessions are indicated in yellow spots and detailed in Table S1. **(B)** Levels of chlorophylls in *Arabidopsis* accessions grown in the presence of 2.5 or 0 μM guazatine for 12 days. Accessions were sorted according to populations and guazatine tolerance. Only populations for which four or more individuals were available are shown. Values are the mean of at least five biological replicates ± SD.

We selected 2.5 μM for the quantitative analysis of the natural variation in response to guazatine in 107 *Arabidopsis* accessions originally collected from worldwide. Higher concentrations were lethal for most natural accessions, whereas 2.5 μM guazatine was optimal to generate a large degree of phenotypic variation (Figures 2A,B and Table S1). We determined total chlorophyll levels as proxy for the quantification of guazatine tolerance traits. Quantification of chlorophyll in guazatine treated and untreated seedlings evidenced the occurrence of quantitative variation for this trait, with some accessions exhibiting high sensitivity and others increased tolerance to the fungicide (Figure 2B). Guazatine resistant and sensitive accessions were evenly distributed in populations from Austria, Czech Republic, France, Germany, The Netherlands, Portugal, UK and USA, with a high frequency of guazatine tolerance in accessions from Germany. We concluded that guazatine tolerant and sensitive accessions are not geographically restricted. Their distributions do not exhibit evident population patterns, although the frequencies of tolerant and sensitive accessions vary between populations (Figure 2B).

### GWAS analysis for chlorophyll levels in response to guazatine

**Figure 3:**
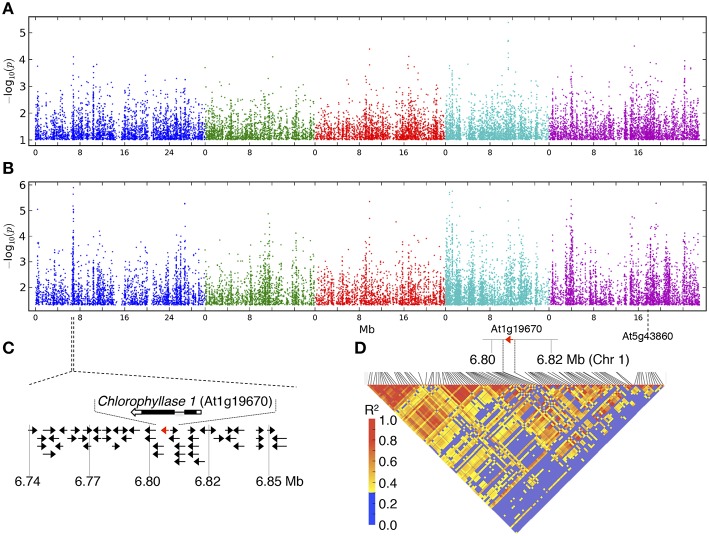
**GWA mapping for guazatine tolerance in 107 *Arabidopsis* accessions**. GWA mapping profiles using the **(A)** accelerated mixed model (AMM) and **(B)** linear regression (LM) methods. Chromosomes are shown in different colors. **(C)** Schematic representation of the *CLH1* flanking region. **(D)** LD triangle plot for the *CLH1* genomic region.

GWAS was conducted to identify genetic factors underlying the response to guazatine in *Arabidopsis* natural populations using chlorophyll levels. The GWAS profiles showed a complex regulation of guazatine tolerance using both the accelerated mixed model (AMM) and linear regression (LM) methods (Figures 3A,B; Seren et al., 2012). Confounding due to population structure between both methods was assessed using Q–Q plots (Figure S1). The AMM method presented lower deviation from the identity line than the LM method, indicating an efficient control for population structure (Figure S1). Several strong associations were identified on the top of chromosome one between 6.74 and 6.87 Mb (Figure 3B) using the LM method. Remarkably, this association was absent under control conditions (Figure S2). The difference between methods seems to be due to the correction for population structure. The risk of *P*-value overcorrection is absent in the LM method when applied to traits correlated with population structure. Considering the advantages and disadvantages of both methods, we investigated potential gene candidates obviously associated with the variation of chlorophyll content within the associated region. *CHLOROPHYLLASE 1* (*CLH1, At1g19670*), involved in the chlorophyll degradation pathway (Hörtensteiner, 2006), is located in the associated region on chromosome one (Figure 3C). Pairwise linkage disequilibrium (LD) between SNPs for this region indicated LD values higher than 0.4 near *CLH1* (Figure 3D), denoting strong LD. The *CLH1* gene has one gene paralog, *CLH2* (*At5g43860*), located on chromosome 5 for which associations could not be detected regardless of the method (Figures 3A,B and Figure S3). We concluded that *CLH1* was an obvious candidate for gene validation studies.

### Characterization of *clh1* and *clh2* mutants in response to guazatine treatment

**Figure 4:**
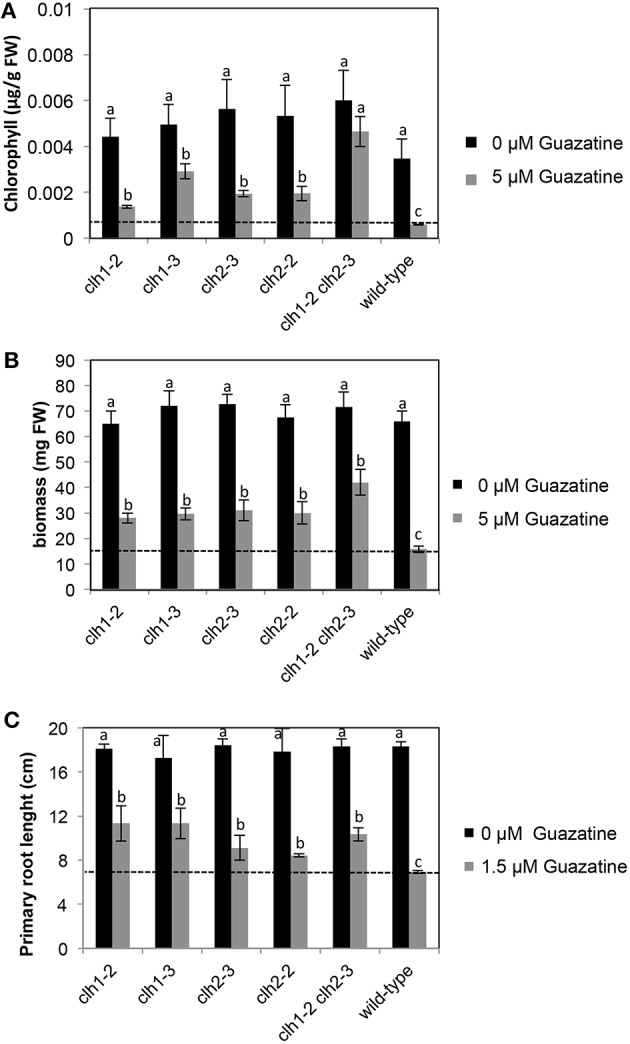
**Chlorophyll levels (A), biomass (B) and primary root length (C) in *clh*, *clh2* and double *clh1 clh2* mutants grown in the presence of 5 or 0 μM guazatine**. Values are the mean of at least five biological replicates ±SD. Letters indicate values that are significantly different according to Student-Newman-Keuls test at *P* < 0.05.

We isolated *clh1* (*clh1-2* and *clh1-3*) and *clh2* (*clh2-2* and *clh2-3*) T-DNA insertion mutants that exhibited reduced or no expression of *CLH1* and *CLH2* genes, respectively, (Figure S4). In agreement with that previously reported for *clh1-1, clh2-1*, and *clh2-2*, mutants (Schenk et al., 2007), *clh1-2, clh1-3*, and *clh2-3* in this work did not show visually evident phenotypes on development or natural senescence differing from wild-type plants. We tested the tolerance of these genotypes to 5 μM guazatine, which is twice the concentration at which most *Arabidopsis* wild-type accessions, including Col-0, exhibited susceptibility (Figure 1B). Remarkably, loss of chlorophyll and growth inhibition induced by 5 μM guazatine treatment was significantly attenuated in *clh1-2, clh1-3, clh2-2*, and *clh2-3* seedlings compared to the wild-type (Figures 4A,B). This indicated that *CLH1* and/or *CLH2* loss-of-function or expression down-regulation enhances guazatine tolerance. The double *clh1-2 clh2-3* mutant exhibited higher chlorophyll and biomass in the presence of guazatine than single *clh1-2, clh1-3*, or *clh2-2, clh2-3* mutants, which is consistent with an additive effect by individual mutations (Figures 4A,B). In the root system, we observed that guazatine concentrations as low as 1.5 μM inhibited primary root elongation in wild-type *Arabidopsis* seedlings and this response was attenuated in *clh1-2 and clh1-3* but not so significantly in *clh2-2 or clh2-3* (Figure 4C and Figure S5). The lower dosage required for root growth inhibition might be due to direct uptake by root cells without the need of transport. The double *clh1-2 clh2-3* responded similarly to *clh1* loss of function (Figure 4C). We concluded that *CLH1* and/or *CLH2* loss-of-function or their down-regulation promote guazatine tolerance in *Arabidopsis*.

### Polyamine levels in response to guazatine treatment

**Figure 5:**
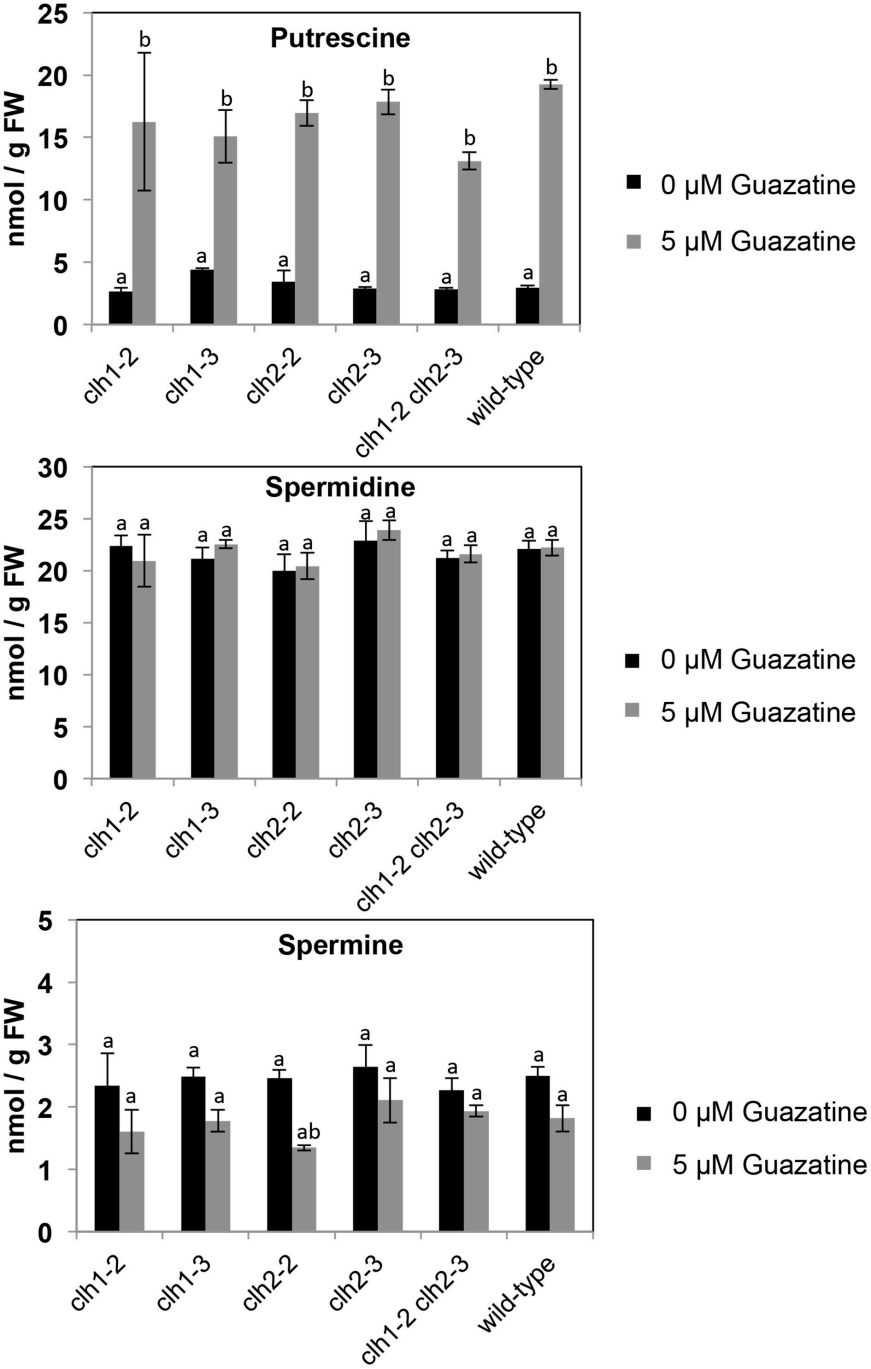
**Levels of free puterscine, spermidine, and spermine in *clh1*, *clh2* and double *clh1 clh2* mutants grown in the presence of 5 or 0 μM guazatine**. Values are the mean of at least five biological replicates ± SD. Letters indicate values that are significantly different according to Student-Newman-Keuls test at *P* < 0.05.

Free putrescine (Put), spermidine (Spd) and spermine (Spm) levels were quantified in *clh1-2, clh1-3, clh2-2, clh2-3*, double *clh1-2 clh2-3* and wild-type seedlings treated or not with 5 μM guazatine during 16 days. Free Put levels accumulated up to 6.7-fold in guazatine-treated seedlings compared to untreated controls (Figure 5). No evident differences in Put levels were apparent between *clh1-2, clh1-3, clh2-2, clh2-3* or double *clh1-2 clh2-3* mutants and the wild-type (Figure 5). The levels of free Spd did not change in response to guazatine treatment, whereas those of free Spm were slightly reduced in all genotypes tested (Figure 5). We concluded that guazatine leads to accumulation of free Put and slight reduction of Spm, and this response was similar in *clh1, clh2* and wild-type plants. Therefore, *CLH1* and *CLH2* mutations do not affect PA responsiveness to guazatine.

### Natural allelic variation at *clh1* and *clh2*

**Figure 6:**
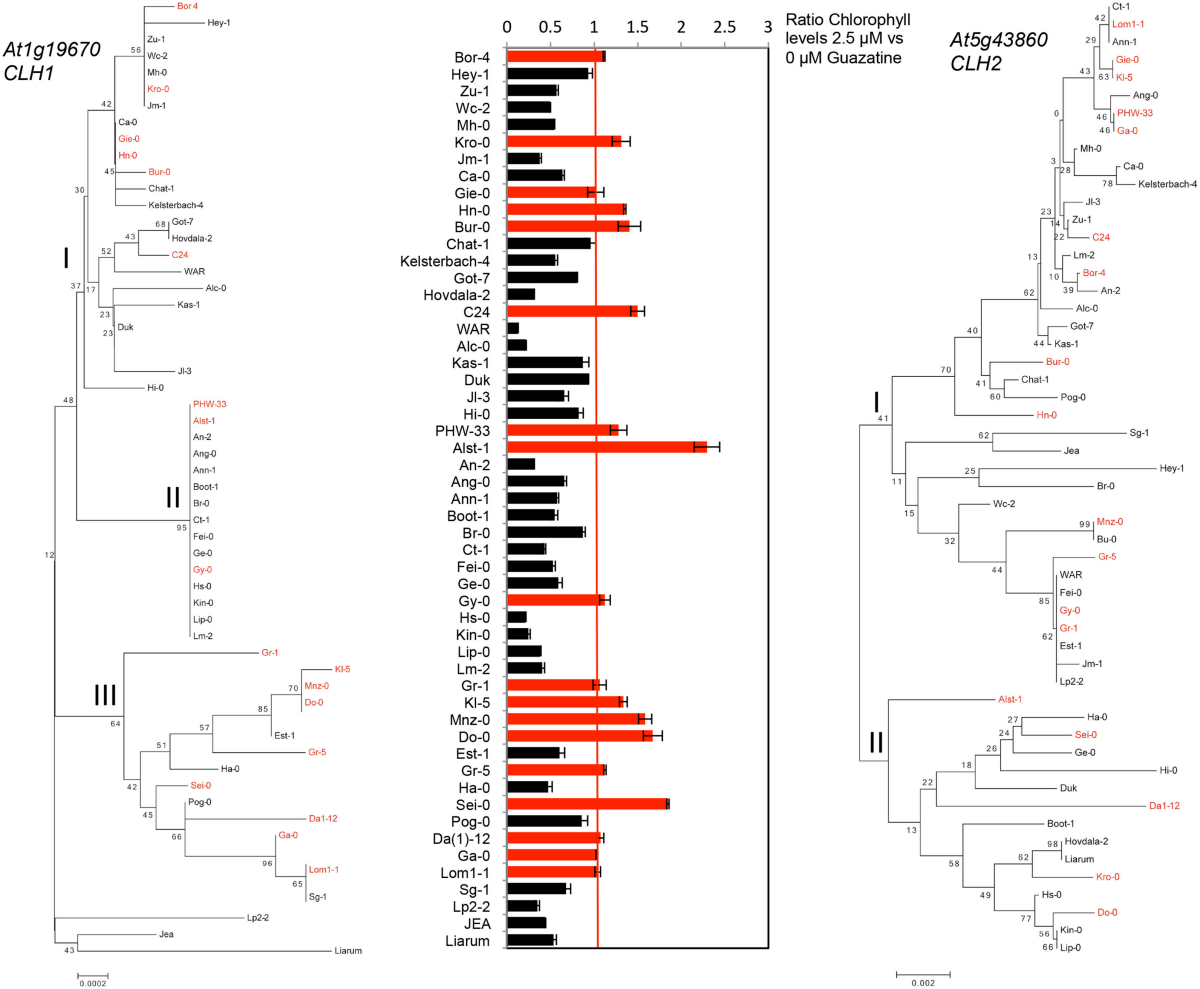
**Neighbor-Joining (NJ) tree of *At1g19670* (*CLH1*) and (*At5g43860*) *CLH2* genes**. Bootstrap values for different nodes are indicated (% from 1000 replicates). Alleles from guazatine-tolerant accessions are highlighted in red.

The sequence of *CLH1* and *CLH2* genes from 53 accessions used in this study (Table S1) was obtained from the 1001 genomes project (www.1001genomes.org) and used to construct *CLH1* and *CLH2* phylogenies (Figure 6). *CLH1* alleles from guazatine tolerant accessions were found in different branches of the *CLH1* tree. However, we observed that 10 out of the 22 guazatine tolerant accessions analyzed in this phylogeny, clustered together in the same branch of the tree (clade III), which indicated that they carry similar *CLH1* alleles. Guazatine tolerant accessions in this clade belonged to populations from Germany (Kl-5, Mnz-0, Do-0, and Ga-0), Austria (Gr-1 and Gr-5), Italy (Sei-0), Czech Republic (Da1-12) and Sweden (Lom1-1). Most guazatine tolerant accessions in this *CLH1* clade did not cluster together in the *CLH2* phylogeny, for which variation was higher (Figure 6). Hence, the *CLH1* clade III was not likely due to simple genetic relationship between accessions, except for Gr-1 and Gr-5. Because of the contribution of both *CLH1* and *CLH2* genes to guazatine tolerance, and the high diversity of *CLH2* alleles detected that does not correlate with *CLH1* phylogeny, we could not identify straightforward associations between specific *CLH1* polymorphism(s) and guazatine tolerance traits by simple comparison between tolerant and sensitive variants.

**Figure 7:**
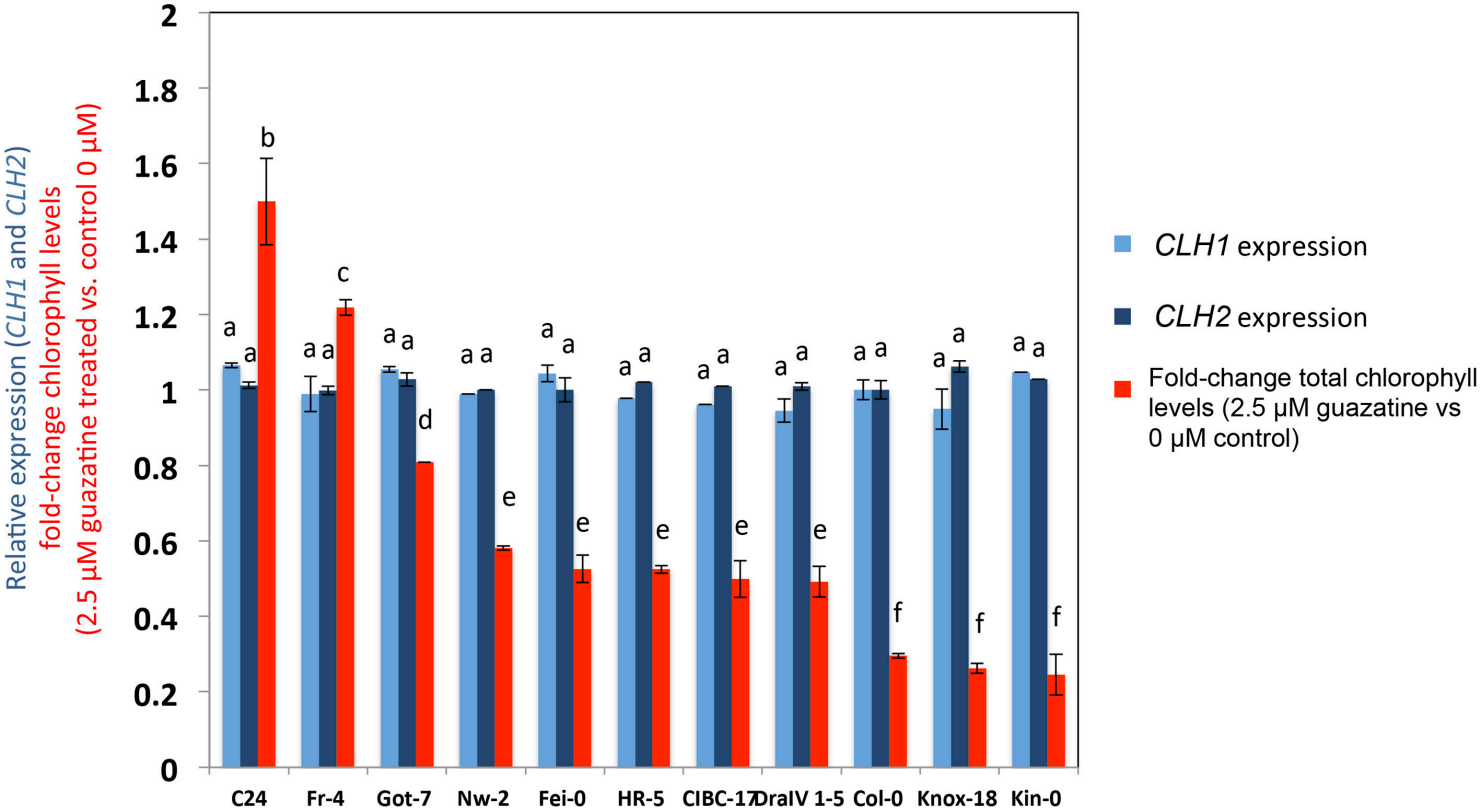
**Gene expression analyses of *CLH1* and *CLH2* and guazatine tolerance**. Expression values in different accessions were obtained from Genevestigator (Hruz et al., 2008). Values were obtained from biological triplicates, normalized to *UBIQUITIN 10* as housekeeping gene, and expressed relative to Col-0 ± SD. Guazatine tolerance was measured by calculating the fold-change of total chlorophyll levels in 2.5 μM guazatine-treated seedlings vs. untreated control (see Materials and Methods). Letters indicate values that are significantly different according to Student-Newman-Keuls test at *P* < 0.05.

*CLH1* and *CLH2* expression was studied in 11 natural accessions that showed contrasted guazatine-tolerance traits (Figure 7). This analysis evidenced the absence of variation in *CLH1* and *CLH2* transcript levels between the accessions. Therefore, changes in *CLH1* and *CLH2* expression are unlikely to underlie guazatine tolerance in *Arabidopsis* natural populations. Rather, we suggest that non-synonymous substitutions in the coding sequence of CLH1 and CLH2 may cause the quantitative variation observed (Figure 7).

Overall, we report that genetic variation at both *CLH1* and *CLH2* genes conditions guazatine tolerance in *Arabidopsis*. The occurrence of multiple (rare) *CLH2* alleles contributing to guazatine tolerance may limit the identification of associations between *CLH2* and guazatine tolerance by GWAS, which was validated by mutant analysis.

## Discussion

In this work, we report the deleterious effects derived from the exposure to low (2.5 μM) concentrations of guazatine in *Arabidopsis* seedlings, the occurrence of extensive natural variation for guazatine tolerance traits in a set of 107 accessions, and the identification of genes involved in this response by GWA mapping. Guazatine is used in agriculture as fungicide recommended for cereals and citrus fruits. We have observed that treatment of the weed *Arabidopsis* with micromolar concentrations of guazatine inhibits growth, primary root elongation and depletes chlorophyll levels (Figures 1, 4). Due to these effects, we conclude that guazatine may be used as herbicide. The 107 accessions selected were sufficient to perform GWA mapping for guazatine tolerance traits and identify *CLH1* as candidate gene (Figure 3C), that together with *CLH2*, were further validated using loss-of-function mutants.

The identification of *CLH* genes underlying guazatine-tolerance traits indicates the involvement of chlorophyll degradation pathways in this response. *Arabidopsis* carries two *CLH* coding genes (*CLH1* and *CLH2*; Benedetti, 1998; Tsuchiya et al., 1999; Benedetti and Arruda, 2002) but associations with guazatine tolerance were only detected for *CLH1* (Figure 3). Because *CLH2* exhibits higher allelic diversity (Figure 6), we reason that the occurrence of multiple, low frequent *CLH2* alleles contributing to guazatine tolerance, might affect the identification of this locus by GWAS. Furthermore, predominant activity of CLH1 over CLH2 in *Arabidopsis* has been reported (Schenk et al., 2007). Interestingly, no variation in *CLH1* and *CLH2* expression was evidenced in *Arabidopsis* accessions differing in their tolerance to guazatine (Figure 7). We suggest that SNPs leading to non-synonymous substitutions in the coding sequence of *CLH1* and *CLH2* may underlie the naturally occurring variation observed, which is compatible with GWAS analysis.

CLH and pheophytinase (PPH) activities catalyze the cleavage of the lipophilic phytol chain of chlorophyll to produce chlorophyllide, a more hydrophilic derivative (Hörtensteiner, 2006). However, the biological assessment of *CLH* function in *Arabidopsis* indicated that *CLH1* and *CLH2* are not involved in senescence-related chlorophyll breakdown (Schenk et al., 2007; Hu et al., 2015). In *Arabidopsis*, PPH is localized in chloroplasts (Schelbert et al., 2009) whereas CLH1 and CLH2 are not plastidial proteins (Schenk et al., 2007). CLH1 is located in the ER and tonoplast of plant cells (Hu et al., 2015). Because of the CLH1 localization, chlorophyll could only be substrate of CLH activity upon release of chlorophyll from the thylakoid membranes. This may be caused by different types of abiotic and biotic stresses that damage plant tissues (Karpinski et al., 2003), or the use of guazatine (Figure 1C). In high amounts, some tetrapyrroles can generate ROS and induce cell death (Kruse et al., 1995; Meskauskiene et al., 2001; Hörtensteiner, 2006; Hirashima et al., 2009). Hu et al. (2015) suggested that CLH and chlorophyll constitute a binary defense system effective against certain chewing herbivores, due to the inducible production of chlorophyllide upon attack, which is toxic for *Spodoptera litura* larvae. Similarly, accumulation of chlorophyllide is a defense mechanism against infection by the necrotrophic fungus *Alternaria brassicicola* in *Arabidopsis* (Kariola et al., 2005). Yagura et al. (1984) reported that the fungal activity of guazatine is due to alterations in membrane integrity, permeability and composition. In *Arabidopsis*, the physiological effects of guazatine application have been less studied. Its use as PAO inhibitor does not require long-term exposure and, for PAO inhibition, guazatine is frequently added to protein extracts for *in vitro* enzymatic reactions. In this work, we report that long-term exposure to guazatine induces membrane damage in *Arabidopsis*, which was evidenced in the alteration of chloroplast integrity followed by chlorophyll degradation. Accumulation of osmophilic bodies, which resembled plastoglobules, was evidenced in guazatine-treated leaves (Figure 1C). Such particles accumulate in response to different stresses and senescence, in parallel to the break-down of thylakoid integrity (Austin et al., 2006). Interestingly, guazatine toxicity is not evident in monocots like oat (Capell et al., 1993). For auxinic herbicides, selectivity between dicots and monocots is due to differences in auxin translocation, degradation, perception, and vascular physiology (Gauvrit and Gaillardon, 1991; Monaco et al., 2002; Kelley and Reichers, 2007). Similar mechanisms may underlie guazatine selectivity between dicots and monocots. However, within-species variation in *Arabidopsis* can be explained by genetic determinisms involving natural variation at *CLH1* and *CLH2* genes.

*CLH1* and/or *CLH2* loss-of-function or expression down-regulation attenuate guazatine toxicity in *Arabidopsis*. We suggest that loss of CLH activity limits chlorophyll degradation under stress conditions that damage the integrity of chloroplast membranes. This would prevent ROS generation and cell death induced by CLH enzymatic activity. Interestingly, no significant differences were observed between the PA profiles of wild-type, *clh1* and *clh2* mutants treated with guazatine. These observations suggest that guazatine effects under long-term exposure of *Arabidopsis* seedlings is not due to its activity as PAO inhibitor, but to other mechanisms involving oxidative stress and/or membrane damage.

PAs in the chloroplast are found as free or conjugated forms, the latter forms produced by transglutaminase activities that bind polyamines to stromal and thylakoid proteins (Kotzabasis et al., 1993; Del Duca et al., 1994; Della Mea et al., 2004; Ioannidis et al., 2009; Hamdani et al., 2011). PAs in the photosynthetic apparatus are beneficial and protect against photoinhibition and ROS production (Navakoudis et al., 2003; Demetriou et al., 2007; Hamdani et al., 2011; Yaakoubi et al., 2014). Surprisingly, guazatine application in osmotically stressed oat leaves resulted beneficial and enhanced Spd and Spm levels, which led to the prevention of chlorophyll loss and senescence (Capell et al., 1993). This contrasts with the effects observed in *Arabidopsis*, in which Put accumulated but Spd or Spm increases were absent (Figure 5). Guazatine application to *Vitis vinifera* also induced Put accumulation with no concomitant changes in the levels of Spd or Spm (Agudelo-Romero et al., 2014). In this case, the raise in Put levels was likely due to activation of ABA pathway and increased expression of *Arginine Decaborxylase (ADC)*, encoding the first biosynthetic step in the ADC pathway to Put biosynthesis (Agudelo-Romero et al., 2014). We conclude that PA profiles by guazatine treatment vary between species, which may be related to the predominance of terminal catabolism and/or PA back-conversion pathways between species.

Overall, we report natural mechanisms by which *Arabidopsis* populations can overcome toxicity by polyaminoguanidine-based fungicides used in agriculture, which might be the result of local adaptation processes.

## Author contributions

KA performed all the experimental research. LB performed the GWAS analyses. KA, AT and RA planned the experiments. RA analyzed the data and wrote the paper with contributions from all authors.

### Conflict of interest statement

The authors declare that the research was conducted in the absence of any commercial or financial relationships that could be construed as a potential conflict of interest. The reviewer, MC and handling Editor declared their shared affiliation, and the handling Editor states that the process nevertheless met the standards of a fair and objective review.

## Supplementary material

Figure S1 **Quantile-Quantile (Q–Q) plots for GWAS analysis of chlorophyll levels in response to guazatine using AMM and LM methods**.

Figure S2 **Genome wide association mapping profile for chlorophyll levels under control conditions (0 μM guazatine) in 107 *Arabidopsis* accessions analyzed with the AMM and LM methods**.

Figure S3 **Detailed view of the genome wide association mapping profile for guazatine tolerance in 107 *Arabidopsis* accessions analyzed with the AMM and LM methods in the *CLH1* (*At1g19670*) and *CLH2* (*At5g43860*) loci**.

Figure S4 **Schematic representation of 5 ′ and 3′ UTRs (white), exons (black), introns (lines), and promoter region (gray) in *CLH1* (*At1g19670*) and *CLH2* (*At5g43860*) genes**. The position of T-DNA insertion in *clh1-2, clh1-3, clh2-2*, and *clh2-3* is indicated. The expression of *CLH1* and *CLH2* in 7-days-old *clh1* and *clh2* seedlings, respectively, was determined by RT-PCR using gene-specific primers and *ACTIN2* as housekeeping control.

Figure S5 **Root phenotype of *clh1-2, clh1-3, clh2-2, clh2-3* and double *clh1-2 clh2-3* 16-days-old seedlings**. Seedlings were germinated and grown in the absence of guazatine during 4 days, and then transferred to vertical plates containing 1.5 μM guazatine. Pictures were taken 12 days after treatment.

Table S1 **List of *Arabidopsis thaliana* accessions used in this work**.
