# Supplementary figures and images for: Genome Wide Association Mapping for the Tolerance to the Polyamine Oxidase Inhibitor Guazatine in Arabidopsis thaliana
Source: Front Plant Sci. 2016 Apr 5;7:401. doi: 10.3389/fpls.2016.00401 (PMC4820465; doi:10.3389/fpls.2016.00401)

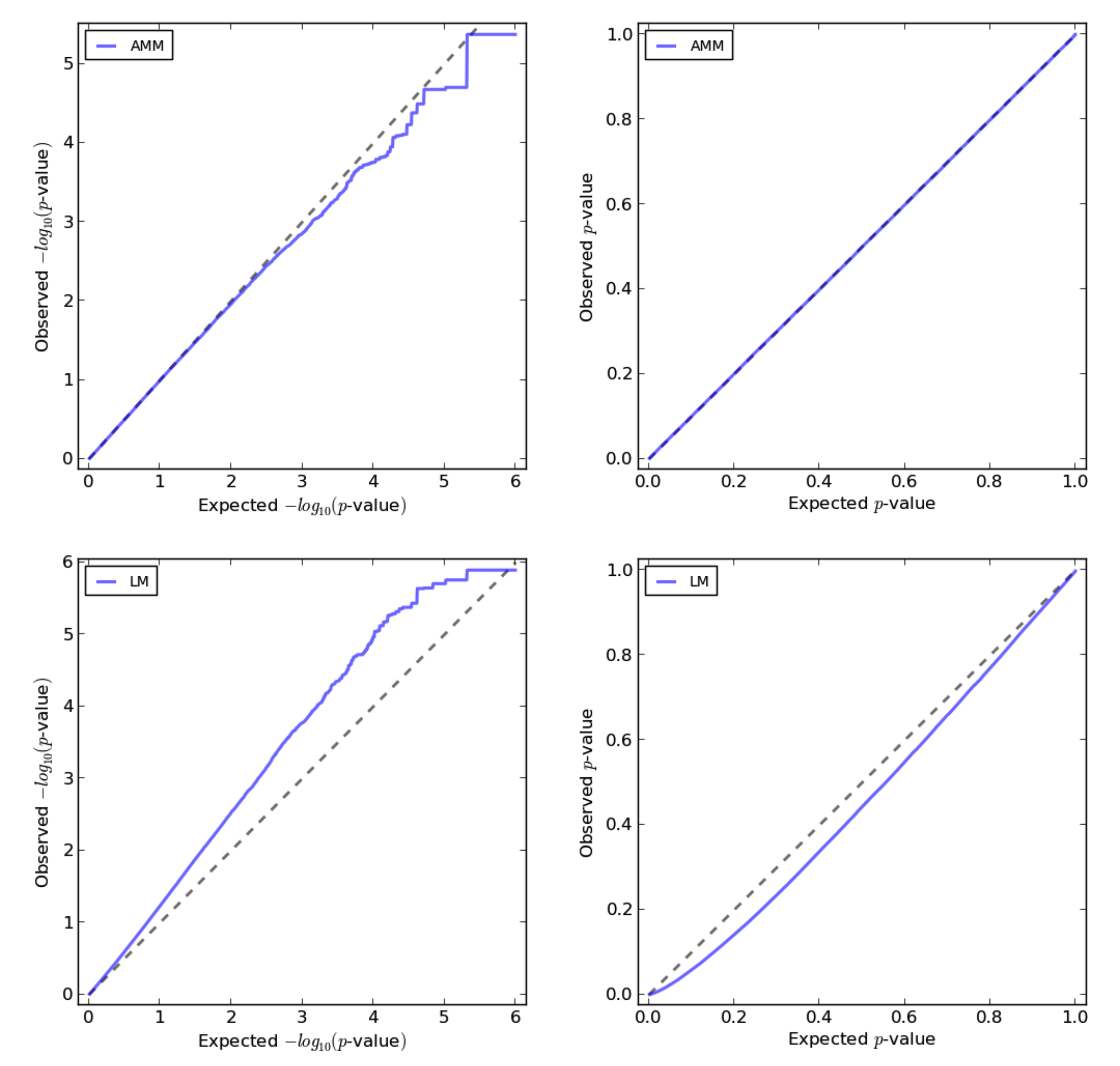

Supplement: Figure S1 — Quantile-Quantile (Q–Q) plots for GWAS analysis of chlorophyll levels in response to guazatine using AMM and LM methods. [file Image1.TIF]

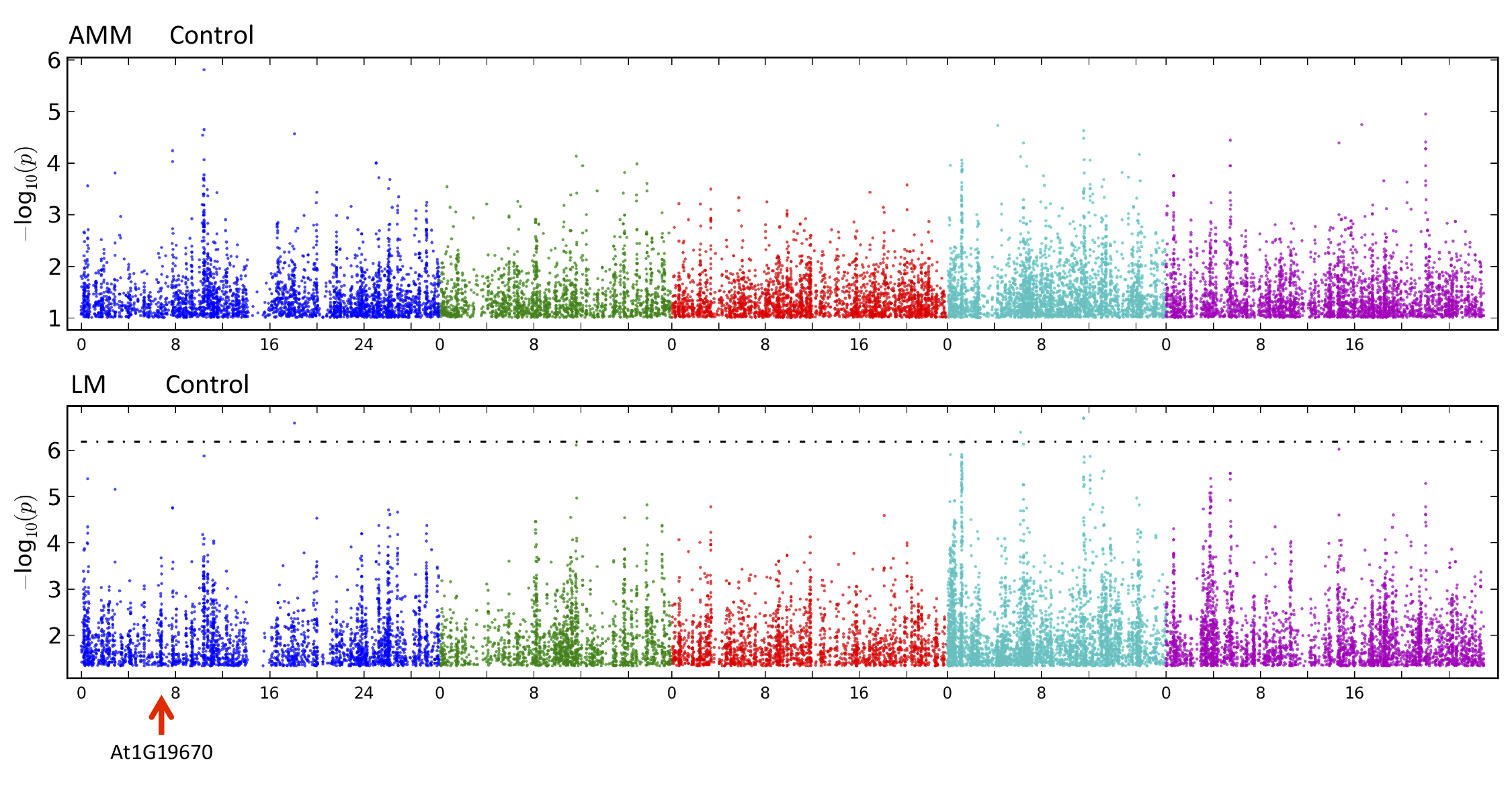

Supplement: Figure S2 — Genome wide association mapping profile for chlorophyll levels under control conditions (0 μM guazatine) in 107 Arabidopsis accessions analyzed with the AMM and LM methods. [file Image2.TIF]

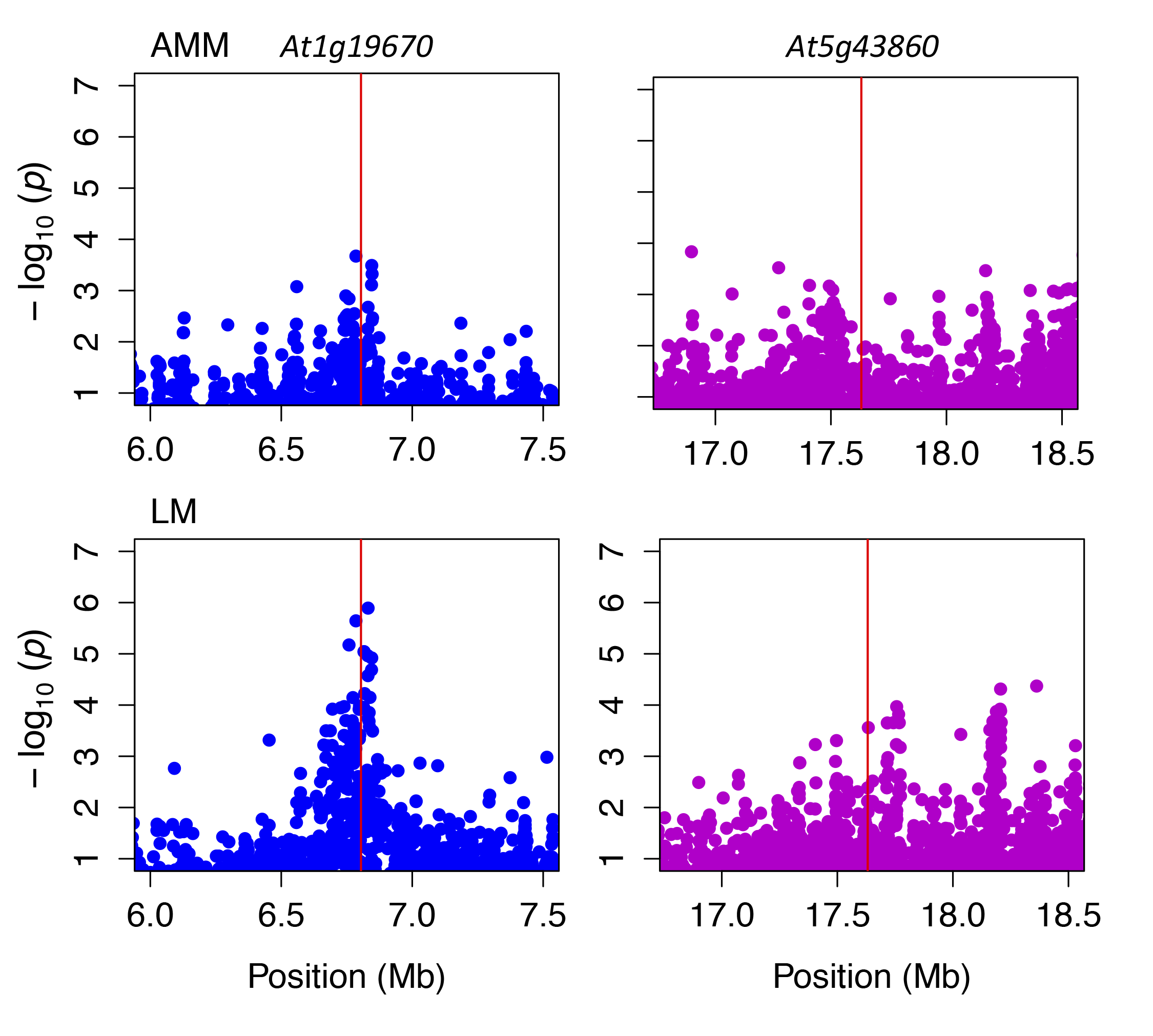

Supplement: Figure S3 — Detailed view of the genome wide association mapping profile for guazatine tolerance in 107 Arabidopsis accessions analyzed with the AMM and LM methods in the CLH1 (At1g19670) and CLH2 (At5g43860) loci. [file Image3.TIF]

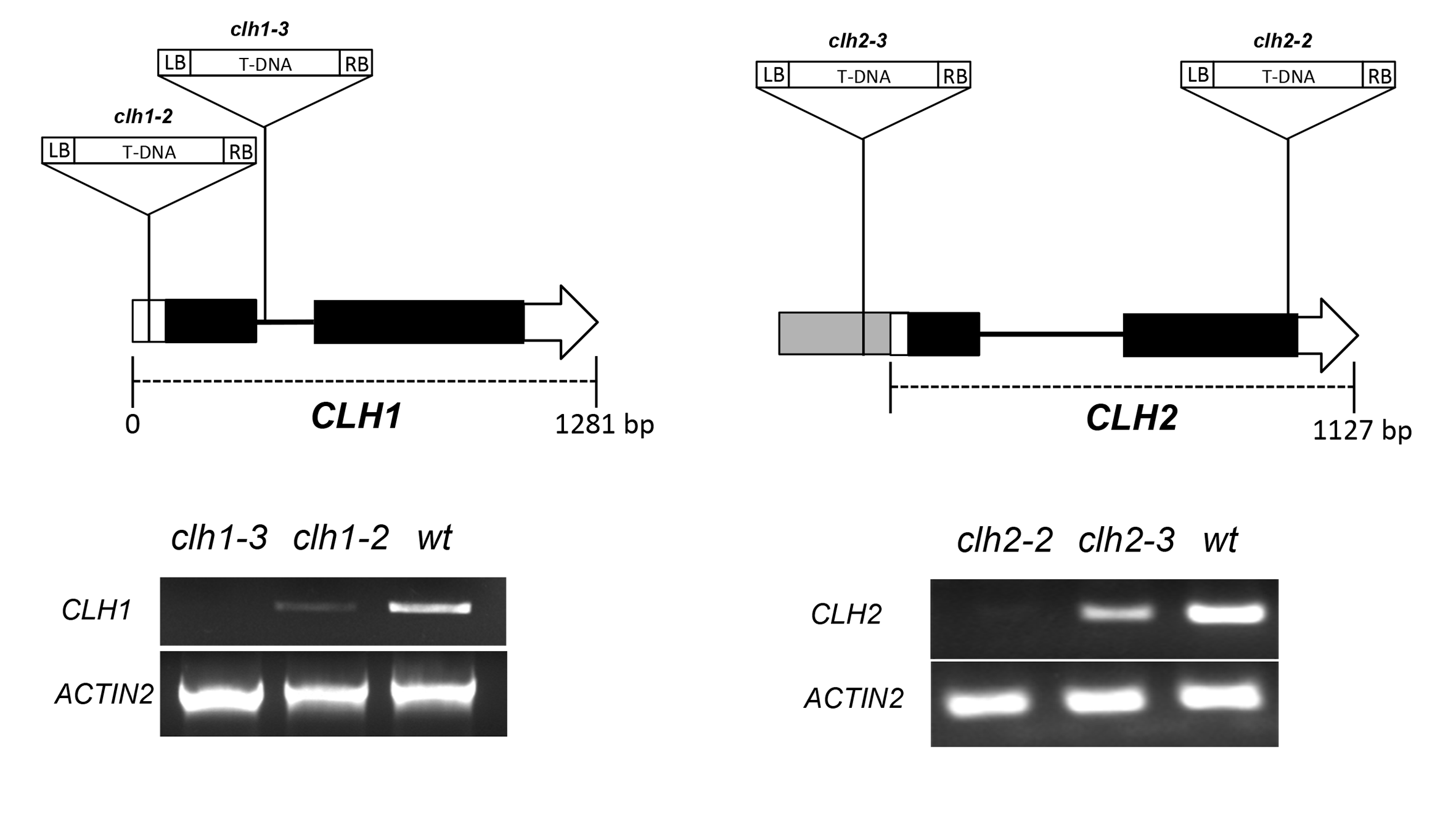

Supplement: Figure S4 — Schematic representation of 5 ′ and 3′ UTRs (white), exons (black), introns (lines), and promoter region (gray) in CLH1 (At1g19670) and CLH2 (At5g43860) genes. The position of T-DNA insertion in clh1-2, clh1-3, clh2-2, and clh2-3 is indicated. The expression of CLH1 and CLH2 in 7-days-old clh1 and clh2 seedlings, respectively, was determined by RT-PCR using gene-specific primers and ACTIN2 as housekeeping control. [file Image4.TIF]

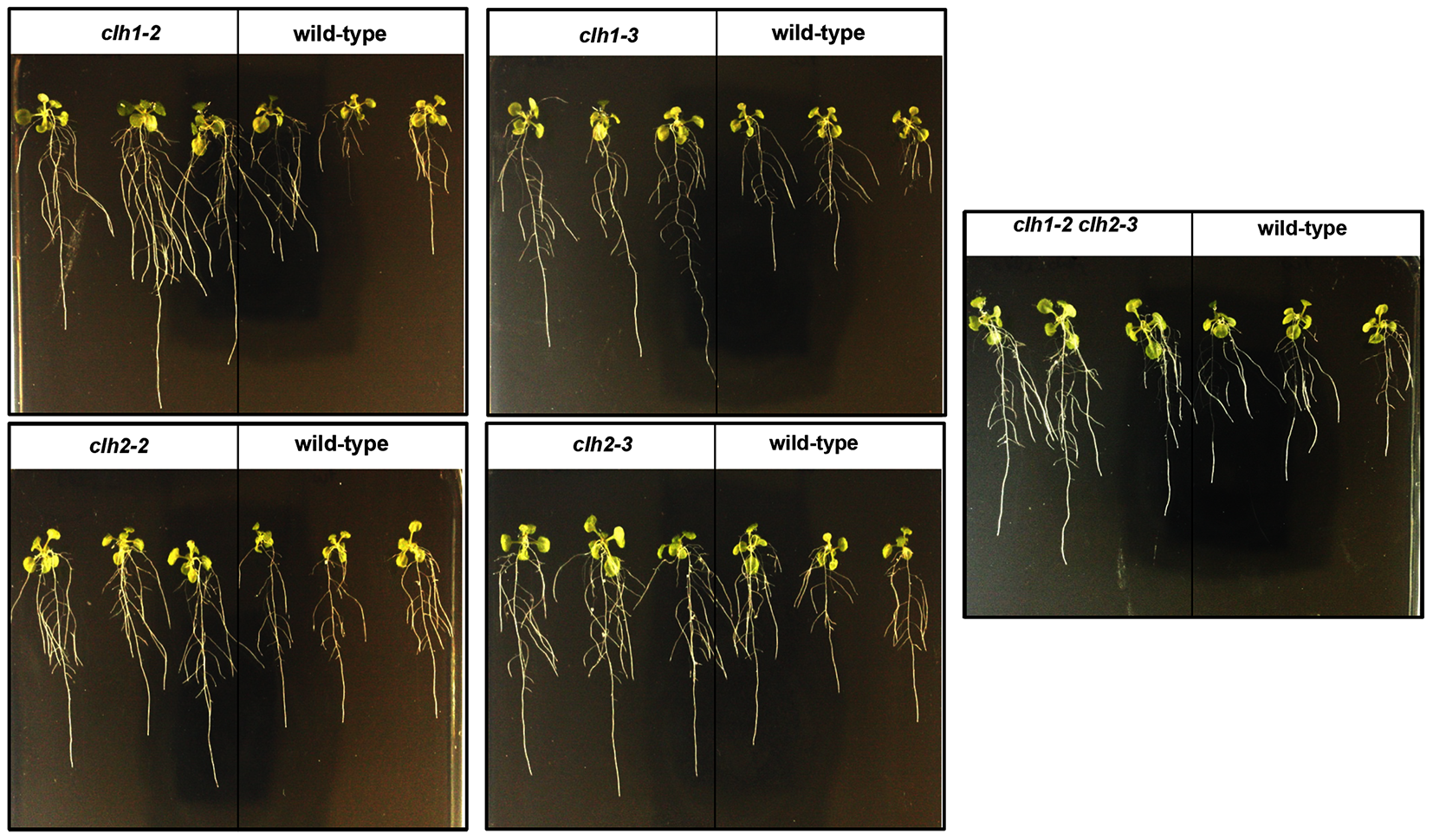

Supplement: Figure S5 — Root phenotype of clh1-2, clh1-3, clh2-2, clh2-3 and double clh1-2 clh2-3 16-days-old seedlings. Seedlings were germinated and grown in the absence of guazatine during 4 days, and then transferred to vertical plates containing 1.5 μM guazatine. Pictures were taken 12 days after treatment. [file Image5.TIF]
